# The low-temperature triclinic crystal structure of silver 3-sulfo­benzoic acid

**DOI:** 10.1107/S2056989020009408

**Published:** 2020-07-14

**Authors:** Reuben T. Bettinger, Philip J. Squattrito, Darpandeep Aulakh

**Affiliations:** a Department of Chemistry and Biochemistry, Central Michigan University, Mount Pleasant, Michigan 48859, USA; b College of Natural Sciences and Mathematics, University of Toledo, Toledo, OH 43606, USA

**Keywords:** crystal structure, silver sulfonate salt, low temperature, hydrogen bonding

## Abstract

Poly[(μ_4_-3-carboxybenzenesulfonato)silver(I)], Ag(O_3_SC_6_H_4_CO_2_H) or [Ag(C_7_H_5_O_5_S)]_*n*_, has been found to undergo a reversible phase transition from monoclinic to triclinic between 160 and 150 K. The low-temperature triclinic structure (space group *P* ) has been determined at 100 K. In contrast to the reported room temperature monoclinic structure, in which the nearly equivalent carboxyl­ate C—O distances indicate that the acidic hydrogen is randomly distributed between the O atoms, at 100 K the C—O (protonated) and C=O (unprotonated) bonds are clearly resolved, resulting in the reduction in symmetry from *C*2/*c* to *P* .

## Abstract 2

Silver(I) 3-carb­oxy­benzene­sulfonate, Ag(O_3_SC_6_H_4_CO_2_H), has been found to undergo a reversible phase transition from monoclinic to triclinic between 160 and 150 K. The low-temperature triclinic structure (space group *P* 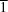) has been determined at 100 K.

## Chemical context   

Over the past two decades, organo­sulfonate and organo­carboxyl­ate anions have received significant attention as building blocks for metal-organic framework (MOF) structures (Dey *et al.*, 2014 ▸; Shimizu *et al.*, 2009 ▸). As a result of its soft nature, sulfonate tends to bond well with soft cations like silver(I) so a significant chemistry of silver sulfonates has developed during this period (Côté & Shimizu, 2004 ▸; Hoffart *et al.*, 2005 ▸). Having previously investigated some structures of silver sulfonate salts (Downer *et al.*, 2006 ▸; Squattrito *et al.*, 2019 ▸), we have continued this effort with the reaction of Ag^+^ with the bifunctional 3-sulfobenzoate anion. The resulting monobasic salt has been found to have an unexpected low-temperature structural modification that is reported here.
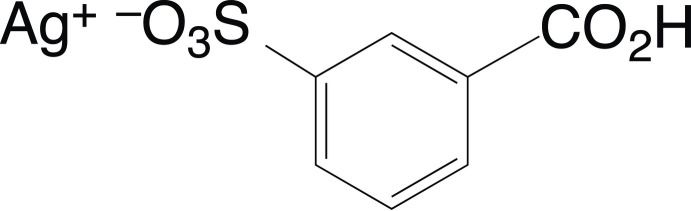


## Structural commentary   

**Figure 1:**
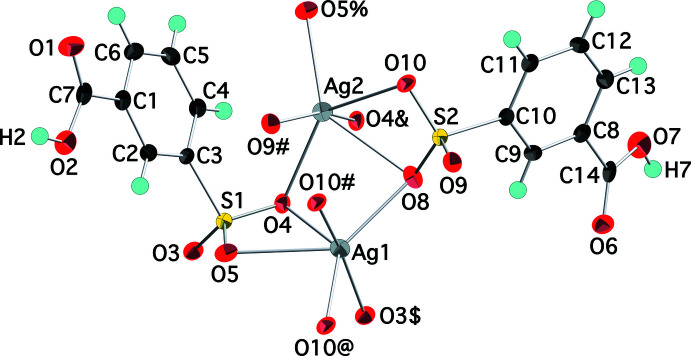
The mol­ecular structure of (I), showing the atom-numbering scheme. Displacement ellipsoids are shown at the 75% probability level and hydrogen atoms are shown as small spheres of arbitrary radii. Symmetry-equivalent oxygen atoms are included to show the complete coordination environments of the cations. [Symmetry codes: (#) 2 − *x*, −*y*, 1 − *z*; (@) *x* + 1, *y*, *z*; ($) 3 − *x*, 1 − *y*, 1 − *z*; (&) 2 − *x*, 1 − *y*, 1 − *z*; (%) *x* − 1, *y*, *z*.]

The product of the reaction of silver nitrate and sodium 3-sulfo­benzoic acid is Ag(O_3_SC_6_H_4_CO_2_H), (I), an anhydrous monobasic silver(I) salt of 3-sulfo­benzoic acid. The room-temperature (293 K) structure of (I) was previously reported in the monoclinic space group *C*2/*c* with one independent cation and anion in the asymmetric unit (Prochniak *et al.*, 2008 ▸). We find the structure at 100 K to be triclinic (*P* 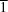) with two independent cations and anions in the asymmetric unit (Fig. 1 ▸). The major features of the structure at 100 K are consistent with those at 293 K. The silver ions are coordinated by six sulfonate O atoms with four shorter (*ca* 2.4–2.5 Å) and two longer (*ca* 2.7 Å) distances (Table 1 ▸) in an irregular hexa­coordinate geometry [somewhat inaccurately described as tetra­hedral by Prochniak *et al.*; the O—Ag—O angles for the four shorter Ag—O bonds range from 71.25 (7) to 164.88 (6)° indicating at best a very distorted tetra­hedron]. Not surprisingly, the Ag—O distances are shorter by an average of 0.02 Å at 100 K than at 293 K. This kind of pseudo-tetra­hedral coordination geometry significantly distorted by two somewhat longer Ag—O inter­actions was previously observed in the silver salt of 6-ammonio­naphthalene-1,3-di­sulfonate (Downer *et al.*, 2006 ▸). The Ag—O distances are consistent with those seen in other silver arene­sulfonates (Côté & Shimizu, 2004 ▸). The extensive metal–sulfonate bonding is as expected given the softer nature of Ag^+^ relative to most *d*-block transition-metal ions (Parr & Pearson, 1983 ▸), which generally show little tendency to bond directly to sulfonate groups (Ma *et al.*, 2003 ▸). The carboxyl­ate group remains protonated with the acidic H atoms unambiguously located on O2 and O7. The C—O distances in the carboxyl­ate groups clearly distinguish the non-protonated (C=O) and protonated (C—O) O atoms: C7—O1 1.232 (3), C7—O2 1.312 (3) Å; C14—O6 1.231 (3), C14—O7 1.311 (3) Å.

## Supra­molecular features   

**Figure 2:**
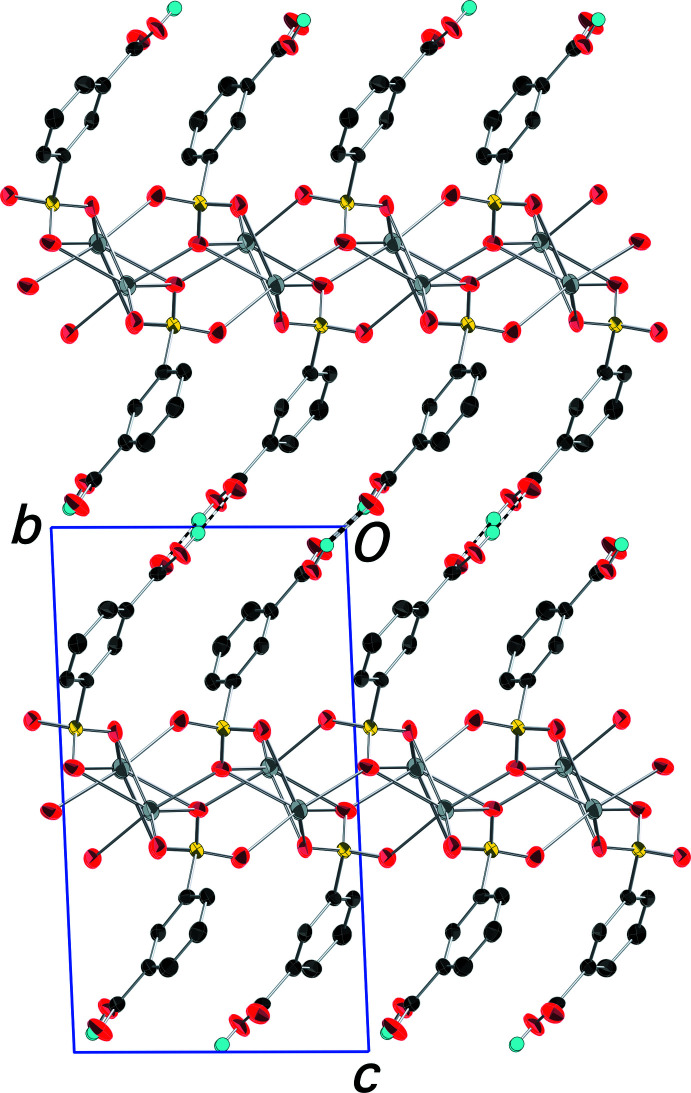
Packing diagram of (I) with an outline of the unit cell. View is onto the (100) plane. The double-layers of 3-sulfo­benzoic acid anions are evident with the silver ions situated in between the layers. O—H⋯O hydrogen bonds connecting the carb­oxy­lic H atoms and carboxyl­ate O atoms of adjacent layers are shown as dashed bonds. H atoms bonded to C atoms have been omitted. Displacement ellipsoids are drawn at the 90% probability level.

**Figure 3:**
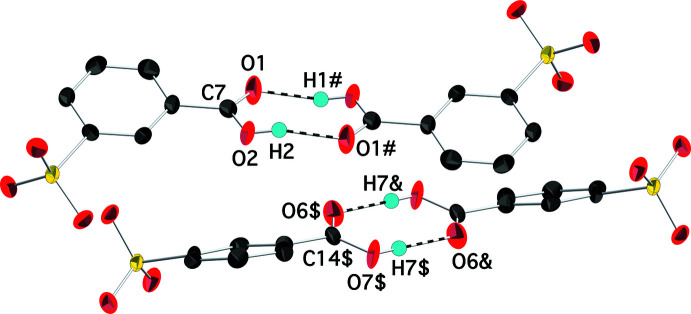
Partial packing diagram of (I) showing the hydrogen-bonding scheme involving the carb­oxy­lic acid groups of neighboring anions. Hydrogen bonds are shown as dashed bonds. Displacement ellipsoids are drawn at the 90% probability level. [Symmetry codes: (#) 2 − *x*, 1 − *y*, 1 − *z*; ($) 3 − *x*, 2 − *y*, 2 − *z*; (&) *x* + 1, *y* + 1, *z*.]

The packing in (I) features layers of metal ions in the *ab* plane alternating with double-layers of 3-sulfo­benzoic acid anions stacking along the *c-*axis direction (Fig. 2 ▸). Anions in adjacent layers are linked by O—H⋯O hydrogen bonds between neighboring carb­oxy­lic acid groups in the classic dimerization of such mol­ecules (Table 2 ▸; Fig. 3 ▸). The symmetry-independent anions alternate in the *b*-axis direction within the layer. The rings of these anions are significantly out of parallel with an inter­planar angle of *ca* 139°. This packing motif with the sulfonate and carboxyl­ate groups directed to opposite sides of the layer is contrary to what was found in the silver salt of the isomeric 4-sulfo­benzoic acid (Squattrito *et al.*, 2019 ▸). In that compound, both functional groups are involved in metal–oxygen bonding so the anions are positioned with both groups equally distributed with respect to each surface of the layer, in contrast to the segregated arrangement in (I).

Comparison of the 100 K and 293 K structures reveals that the key difference is in the carboxyl­ate group. At 293 K, the C—O bond lengths are almost the same [1.250 (3) and 1.271 (3) Å], indicating significant disorder between the protonated and non-protonated O atoms, while at 100 K the C—O and C=O bonds are clearly distinguished and the placement of the acidic H atoms accordingly renders the two 3-sulfo­benzoic acid moieties symmetry-inequivalent. Variable-temperature single-crystal X-ray measurements between 250 and 130 K show that the monoclinic-to-triclinic transition occurs on going from 160 to 150 K and that it is reversible.

## Database survey   

A search of the Cambridge Structural Database (CSD, Version 5.41, update of November 2019; Groom *et al.*, 2016 ▸) for metal 3-sulfobenzoate salts that do not contain aromatic rings containing nitro­gen (aromatic amines are popular secondary linkers in MOF systems) yielded twenty hits. Of these, eleven contain other amines. The nine reported structures containing only metal ions and 3-sulfobenzoate ions (protonated or unprotonated), with or without water mol­ecules, are the 293 K structure of (I) (refcode ROJJUW; Prochniak *et al.*, 2008 ▸), sodium 3-sulfo­benzoic acid dihydrate (ROJJOQ; Prochniak *et al.*, 2008 ▸), disilver disodium bis­(3-sulfobenzoate) hepta­hydrate (EKOXUY; Zheng & Zhu, 2011 ▸), bis­muth(III) 3-sulfo­benzoic acid tetra­hydrate (LEXKAD; Senevirathna *et al.*, 2018 ▸), barium 3-sulfo­benzoic acid trihydrate (FOBXUQ; Gao *et al.*, 2005 ▸), and four mixed 3-sulfobenzoate hydroxo salts of the trivalent lanthanide ions neodymium (UQOYAB; Ying *et al.*, 2010 ▸), europium (EQUBOI; Li *et al.*, 2010 ▸), gadolinium (EQUBUO; Li *et al.*, 2010 ▸), and terbium (EQUBIC; Li *et al.*, 2010 ▸). All of these structures feature direct bonding between the sulfonate O atoms and the metal ions with resulting frameworks of varying dimensionalities.

## Synthesis and crystallization   

A 2.24 g (10.0 mmol) sample of sodium 3-sulfo­benzoic acid (Aldrich, 97%) was dissolved in 45 ml of water. To this colorless solution was added a colorless solution of 1.69 g (9.95 mmol) of AgNO_3_ (Baker) in 45 ml of water. The resulting clear colorless solution was stirred for about 30 minutes and transferred to a porcelain evaporating dish that was set out to evaporate in a fume hood. After several days, the water had completely evaporated leaving behind small colorless needle-shaped crystals, 0.75 g of which were collected by hand from the dish. These were identified as (I) through the single crystal X-ray study.

## Refinement   

Crystal data, data collection and structure refinement details are summarized in Table 3 ▸. Hydrogen atoms bonded to carbon atoms and the carb­oxy­lic hydrogen atoms were located in difference electron-density maps, refined isotropically to confirm their placement, and finally, owing to the presence of the heavy atoms, constrained on idealized positions and included in the refinement as riding atoms with C—H = 0.95 Å or O—H = 0.84 Å and their *U*_iso_ constrained to be 1.2 (C—H) or 1.5 (O—H) times the *U*_eq_ of the bonding atom. There are four relatively large peaks (1.22–1.46 e Å^−3^) in the final difference electron-density map that are located *ca* 0.9 Å on either side of the Ag atoms along the *a* axis. Attempted refinement of the extinction parameter resulted in a value near zero so it was not included in the final model. Although we cannot rule out an issue with the absorption correction, none is evident and the structure is otherwise well-behaved. The variable-temperature single crystal X-ray experiment was done by cooling in 10 K increments from 250 to 130 K and then heating back to 170 K. At each step once the desired temperature was reached, the crystal was maintained at that temperature for 15 minutes before data acquisition. A complete data collection and refinement were also conducted at 296 K to confirm the reported monoclinic structure (Prochniak *et al.*, 2008 ▸). Our results were essentially identical to the reported ones so they are not included here.

## Supplementary Material

Crystal structure: contains datablock(s) I, global. DOI: 10.1107/S2056989020009408/mw2166sup1.cif

Structure factors: contains datablock(s) I. DOI: 10.1107/S2056989020009408/mw2166Isup2.hkl

CCDC reference: 2015332

Additional supporting information:  crystallographic information; 3D view; checkCIF report

## Figures and Tables

**Table 1:** Selected bond lengths (Å)

Ag1—O3^i^	2.3868 (18)	Ag2—O9^ii^	2.4090 (17)
Ag1—O8	2.4091 (18)	Ag2—O5^iv^	2.4199 (18)
Ag1—O10^ii^	2.4406 (18)	Ag2—O4^v^	2.4609 (18)
Ag1—O10^iii^	2.5249 (18)	Ag2—O4	2.5295 (18)
Ag1—O5	2.6853 (19)	Ag2—O8	2.6953 (19)
Ag1—O4	2.7254 (19)	Ag2—O10	2.7179 (19)

Symmetry codes: (i) 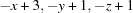; (ii) 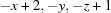; (iii) 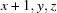; (iv) 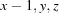; (v) 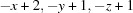.

**Table 2:** Hydrogen-bond geometry (Å, °)

*D*—H⋯*A*	*D*—H	H⋯*A*	*D*⋯*A*	*D*—H⋯*A*
O2—H2⋯O1^vi^	0.84	1.81	2.631 (3)	164
O7—H7⋯O6^vii^	0.84	1.81	2.651 (3)	176

Symmetry codes: (vi) 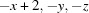; (vii) 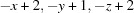.

**Table 3:** Experimental details

Crystal data	
Chemical formula	[Ag(C_7_H_5_O_5_S)]
*M*_r_	309.04
Crystal system, space group	Triclinic, *P* 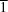
Temperature (K)	100
*a*, *b*, *c* (Å)	6.0376 (5), 8.6293 (7), 15.5903 (12)
α, β, γ (°)	92.315 (1), 99.589 (1), 90.657 (1)
*V* (Å^3^)	800.12 (11)
*Z*	4
Radiation type	Mo *K*α
μ (mm^−1^)	2.77
Crystal size (mm)	0.10 × 0.09 × 0.02

Data collection	
Diffractometer	Bruker APEXII CCD
Absorption correction	Multi-scan (*SADABS*; Krause *et al.*, 2015 ▸)
*T*_min_, *T*_max_	0.675, 0.746
No. of measured, independent and observed [*I* > 2σ(*I*)] reflections	11343, 3990, 3464
*R*_int_	0.019
(sin θ/λ)_max_ (Å^−1^)	0.668

Refinement	
*R*[*F* ^2^ > 2σ(*F* ^2^)], *wR*(*F* ^2^), *S*	0.023, 0.057, 1.02
No. of reflections	3990
No. of parameters	255
H-atom treatment	H-atom parameters constrained
Δρ_max_, Δρ_min_ (e Å^−3^)	1.46, −0.54

Computer programs: *APEX3* and *SAINT* (Bruker, 2015 ▸), *SHELXT2014/5* (Sheldrick, 2015*a* ▸), *SHELXL2018/3* (Sheldrick, 2015*b* ▸) and *CrystalMaker* (Palmer, 2014 ▸).

## supplementary crystallographic information

### Crystal data

**Table:**

[Ag(C_7_H_5_O_5_S)]	*Z* = 4
*M**_r_* = 309.04	*F*(000) = 600
Triclinic, *P*1	*D*_x_ = 2.565 Mg m^−^^3^
*a* = 6.0376 (5) Å	Mo *K*α radiation, λ = 0.71073 Å
*b* = 8.6293 (7) Å	Cell parameters from 5814 reflections
*c* = 15.5903 (12) Å	θ = 2.7–28.4°
α = 92.315 (1)°	µ = 2.77 mm^−^^1^
β = 99.589 (1)°	*T* = 100 K
γ = 90.657 (1)°	Block, colorless
*V* = 800.12 (11) Å^3^	0.10 × 0.09 × 0.02 mm

### Data collection

**Table:**

Bruker APEXII CCD diffractometer	3464 reflections with *I* > 2σ(*I*)
ω and φ scans	*R*_int_ = 0.019
Absorption correction: multi-scan (SADABS; Krause *et al.*, 2015)	θ_max_ = 28.4°, θ_min_ = 2.4°
*T*_min_ = 0.675, *T*_max_ = 0.746	*h* = −8→8
11343 measured reflections	*k* = −11→11
3990 independent reflections	*l* = −20→20

### Refinement

**Table:**

Refinement on *F*^2^	Primary atom site location: dual
Least-squares matrix: full	Secondary atom site location: difference Fourier map
*R*[*F*^2^ > 2σ(*F*^2^)] = 0.023	Hydrogen site location: difference Fourier map
*wR*(*F*^2^) = 0.057	H-atom parameters constrained
*S* = 1.02	*w* = 1/[σ^2^(*F*_o_^2^) + (0.0312*P*)^2^ + 0.7957*P*] where *P* = (*F*_o_^2^ + 2*F*_c_^2^)/3
3990 reflections	(Δ/σ)_max_ = 0.002
255 parameters	Δρ_max_ = 1.46 e Å^−^^3^
0 restraints	Δρ_min_ = −0.54 e Å^−^^3^

### Special details

**Table:**

Geometry. All esds (except the esd in the dihedral angle between two l.s. planes) are estimated using the full covariance matrix. The cell esds are taken into account individually in the estimation of esds in distances, angles and torsion angles; correlations between esds in cell parameters are only used when they are defined by crystal symmetry. An approximate (isotropic) treatment of cell esds is used for estimating esds involving l.s. planes.

### Fractional atomic coordinates and isotropic or equivalent isotropic displacement parameters (Å^2^)

**Table:**

	*x*	*y*	*z*	*U*_iso_*/*U*_eq_	
Ag1	1.38401 (3)	0.20362 (2)	0.54061 (2)	0.01134 (6)	
Ag2	0.84468 (3)	0.29410 (2)	0.45908 (2)	0.01133 (6)	
S1	1.30827 (10)	0.44688 (7)	0.38473 (4)	0.00764 (12)	
S2	0.91114 (10)	0.05323 (7)	0.61650 (4)	0.00735 (12)	
O1	0.8095 (3)	0.0907 (2)	0.04187 (13)	0.0171 (4)	
O2	1.1825 (3)	0.1223 (2)	0.08315 (12)	0.0147 (4)	
H2	1.186373	0.069042	0.037146	0.022*	
O3	1.4157 (3)	0.5928 (2)	0.36958 (12)	0.0118 (4)	
O4	1.1960 (3)	0.4580 (2)	0.46182 (12)	0.0113 (4)	
O5	1.4577 (3)	0.3141 (2)	0.38907 (12)	0.0120 (4)	
O6	1.0685 (3)	0.3705 (2)	0.92789 (12)	0.0151 (4)	
O7	0.7208 (3)	0.4338 (2)	0.94908 (13)	0.0172 (4)	
H7	0.792105	0.496134	0.986827	0.026*	
O8	1.0577 (3)	0.1870 (2)	0.61036 (12)	0.0122 (4)	
O9	1.0299 (3)	−0.0917 (2)	0.63142 (12)	0.0113 (4)	
O10	0.7283 (3)	0.0403 (2)	0.54050 (12)	0.0121 (4)	
C1	0.9468 (4)	0.2658 (3)	0.16077 (16)	0.0098 (5)	
C2	1.1255 (4)	0.3040 (3)	0.22732 (16)	0.0089 (5)	
H2A	1.269323	0.261007	0.226531	0.011*	
C3	1.0903 (4)	0.4059 (3)	0.29493 (16)	0.0085 (5)	
C4	0.8808 (4)	0.4739 (3)	0.29484 (17)	0.0098 (5)	
H4	0.858595	0.544361	0.340934	0.012*	
C5	0.7059 (4)	0.4378 (3)	0.22695 (17)	0.0113 (5)	
H5	0.564694	0.485685	0.225963	0.014*	
C6	0.7358 (4)	0.3320 (3)	0.16042 (17)	0.0122 (5)	
H6	0.614232	0.304894	0.115096	0.015*	
C7	0.9731 (4)	0.1514 (3)	0.08975 (17)	0.0114 (5)	
C8	0.7557 (4)	0.2413 (3)	0.84027 (16)	0.0101 (5)	
C9	0.8758 (4)	0.1989 (3)	0.77403 (16)	0.0089 (5)	
H9	1.022664	0.239791	0.774347	0.011*	
C10	0.7759 (4)	0.0954 (3)	0.70750 (16)	0.0083 (5)	
C11	0.5641 (4)	0.0292 (3)	0.70888 (16)	0.0094 (5)	
H11	0.498762	−0.043294	0.664035	0.011*	
C12	0.4502 (4)	0.0705 (3)	0.77651 (17)	0.0109 (5)	
H12	0.307895	0.024014	0.778523	0.013*	
C13	0.5423 (4)	0.1791 (3)	0.84132 (17)	0.0116 (5)	
H13	0.460534	0.210509	0.885891	0.014*	
C14	0.8629 (4)	0.3547 (3)	0.91029 (16)	0.0104 (5)	

### Atomic displacement parameters (Å^2^)

**Table:**

	*U*^11^	*U*^22^	*U*^33^	*U*^12^	*U*^13^	*U*^23^
Ag1	0.01230 (10)	0.00897 (10)	0.01265 (10)	−0.00049 (7)	0.00235 (7)	−0.00154 (7)
Ag2	0.01222 (10)	0.00908 (10)	0.01249 (10)	0.00046 (7)	0.00190 (7)	−0.00134 (7)
S1	0.0092 (3)	0.0059 (3)	0.0076 (3)	0.0002 (2)	0.0012 (2)	−0.0016 (2)
S2	0.0092 (3)	0.0058 (3)	0.0071 (3)	−0.0001 (2)	0.0017 (2)	−0.0015 (2)
O1	0.0147 (10)	0.0189 (10)	0.0160 (10)	0.0011 (7)	0.0001 (8)	−0.0103 (8)
O2	0.0149 (9)	0.0163 (10)	0.0122 (9)	0.0022 (7)	0.0015 (7)	−0.0069 (7)
O3	0.0131 (9)	0.0091 (9)	0.0124 (9)	−0.0021 (7)	−0.0005 (7)	0.0006 (7)
O4	0.0133 (9)	0.0128 (9)	0.0079 (8)	−0.0011 (7)	0.0025 (7)	−0.0014 (7)
O5	0.0121 (9)	0.0079 (8)	0.0151 (9)	0.0026 (7)	−0.0003 (7)	−0.0016 (7)
O6	0.0154 (10)	0.0156 (10)	0.0134 (9)	−0.0029 (7)	0.0011 (7)	−0.0046 (7)
O7	0.0188 (10)	0.0173 (10)	0.0150 (10)	−0.0009 (8)	0.0041 (8)	−0.0104 (8)
O8	0.0161 (9)	0.0067 (8)	0.0151 (9)	−0.0025 (7)	0.0070 (7)	−0.0017 (7)
O9	0.0132 (9)	0.0087 (8)	0.0128 (9)	0.0037 (7)	0.0049 (7)	−0.0006 (7)
O10	0.0126 (9)	0.0138 (9)	0.0090 (9)	0.0011 (7)	−0.0004 (7)	−0.0025 (7)
C1	0.0139 (12)	0.0069 (11)	0.0087 (11)	0.0001 (9)	0.0025 (9)	−0.0011 (9)
C2	0.0098 (12)	0.0071 (11)	0.0098 (11)	0.0011 (9)	0.0016 (9)	0.0001 (9)
C3	0.0093 (11)	0.0087 (11)	0.0071 (11)	−0.0002 (9)	0.0005 (9)	0.0006 (9)
C4	0.0124 (12)	0.0075 (11)	0.0096 (11)	−0.0002 (9)	0.0023 (9)	−0.0010 (9)
C5	0.0097 (12)	0.0105 (12)	0.0143 (12)	0.0020 (9)	0.0034 (9)	0.0018 (10)
C6	0.0125 (12)	0.0112 (12)	0.0119 (12)	−0.0018 (9)	−0.0004 (9)	0.0003 (9)
C7	0.0170 (13)	0.0092 (11)	0.0083 (12)	0.0014 (9)	0.0023 (9)	0.0011 (9)
C8	0.0127 (12)	0.0082 (11)	0.0087 (12)	0.0007 (9)	0.0000 (9)	−0.0002 (9)
C9	0.0087 (11)	0.0064 (11)	0.0112 (12)	−0.0002 (9)	0.0004 (9)	0.0012 (9)
C10	0.0097 (12)	0.0080 (11)	0.0072 (11)	0.0019 (9)	0.0018 (9)	−0.0015 (9)
C11	0.0117 (12)	0.0069 (11)	0.0091 (11)	0.0006 (9)	0.0005 (9)	0.0003 (9)
C12	0.0082 (11)	0.0112 (12)	0.0133 (12)	−0.0001 (9)	0.0020 (9)	0.0002 (9)
C13	0.0120 (12)	0.0131 (12)	0.0097 (12)	0.0013 (9)	0.0021 (9)	−0.0006 (9)
C14	0.0161 (13)	0.0093 (11)	0.0061 (11)	0.0004 (9)	0.0026 (9)	0.0004 (9)

### Geometric parameters (Å, º)

**Table:**

Ag1—O3^i^	2.3868 (18)	O7—C14	1.311 (3)
Ag1—O8	2.4091 (18)	O7—H7	0.8400
Ag1—O10^ii^	2.4406 (18)	C1—C2	1.393 (3)
Ag1—O10^iii^	2.5249 (18)	C1—C6	1.402 (4)
Ag1—O5	2.6853 (19)	C1—C7	1.483 (3)
Ag1—O4	2.7254 (19)	C2—C3	1.390 (3)
Ag2—O9^ii^	2.4090 (17)	C2—H2A	0.9500
Ag2—O5^iv^	2.4199 (18)	C3—C4	1.400 (3)
Ag2—O4^v^	2.4609 (18)	C4—C5	1.388 (4)
Ag2—O4	2.5295 (18)	C4—H4	0.9500
Ag2—O8	2.6953 (19)	C5—C6	1.389 (4)
Ag2—O10	2.7179 (19)	C5—H5	0.9500
S1—O3	1.4556 (18)	C6—H6	0.9500
S1—O5	1.4629 (18)	C8—C13	1.393 (4)
S1—O4	1.4754 (18)	C8—C9	1.397 (3)
S1—C3	1.776 (3)	C8—C14	1.494 (3)
S2—O9	1.4560 (18)	C9—C10	1.393 (3)
S2—O8	1.4624 (18)	C9—H9	0.9500
S2—O10	1.4784 (19)	C10—C11	1.399 (3)
S2—C10	1.778 (2)	C11—C12	1.388 (3)
O1—C7	1.232 (3)	C11—H11	0.9500
O2—C7	1.312 (3)	C12—C13	1.390 (4)
O2—H2	0.8400	C12—H12	0.9500
O6—C14	1.231 (3)	C13—H13	0.9500
			
O3^i^—Ag1—O8	99.00 (6)	Ag1^i^—O3—Ag2^v^	67.35 (4)
O3^i^—Ag1—O10^ii^	164.88 (6)	S1—O3—Ag2^iii^	68.22 (7)
O8—Ag1—O10^ii^	89.93 (6)	Ag1^i^—O3—Ag2^iii^	91.87 (5)
O3^i^—Ag1—O10^iii^	93.71 (6)	Ag2^v^—O3—Ag2^iii^	105.51 (5)
O8—Ag1—O10^iii^	134.17 (6)	S1—O4—Ag2^v^	122.51 (10)
O10^ii^—Ag1—O10^iii^	71.42 (7)	S1—O4—Ag2	117.62 (10)
O3^i^—Ag1—O5	95.65 (6)	Ag2^v^—O4—Ag2	108.75 (7)
O8—Ag1—O5	133.72 (6)	S1—O4—Ag1	97.01 (9)
O10^ii^—Ag1—O5	86.75 (6)	Ag2^v^—O4—Ag1	123.21 (7)
O10^iii^—Ag1—O5	87.86 (6)	Ag2—O4—Ag1	80.66 (5)
O3^i^—Ag1—O4	79.09 (6)	S1—O4—Ag1^i^	68.13 (7)
O8—Ag1—O4	87.10 (6)	Ag2^v^—O4—Ag1^i^	59.17 (4)
O10^ii^—Ag1—O4	113.75 (6)	Ag2—O4—Ag1^i^	164.54 (7)
O10^iii^—Ag1—O4	138.66 (6)	Ag1—O4—Ag1^i^	113.67 (5)
O5—Ag1—O4	53.11 (5)	S1—O5—Ag2^iii^	129.94 (11)
O9^ii^—Ag2—O5^iv^	100.47 (6)	S1—O5—Ag1	99.04 (9)
O9^ii^—Ag2—O4^v^	163.71 (6)	Ag2^iii^—O5—Ag1	81.60 (5)
O5^iv^—Ag2—O4^v^	88.30 (6)	C14—O7—H7	109.5
O9^ii^—Ag2—O4	92.99 (6)	S2—O8—Ag1	129.24 (10)
O5^iv^—Ag2—O4	134.11 (6)	S2—O8—Ag2	98.80 (9)
O4^v^—Ag2—O4	71.25 (7)	Ag1—O8—Ag2	83.46 (6)
O9^ii^—Ag2—O8	95.18 (6)	S2—O9—Ag2^ii^	135.66 (11)
O5^iv^—Ag2—O8	135.75 (6)	S2—O9—Ag1^ii^	79.15 (8)
O4^v^—Ag2—O8	87.79 (6)	Ag2^ii^—O9—Ag1^ii^	68.49 (4)
O4—Ag2—O8	85.39 (6)	S2—O9—Ag1	68.85 (7)
O9^ii^—Ag2—O10	79.91 (6)	Ag2^ii^—O9—Ag1	90.55 (5)
O5^iv^—Ag2—O10	89.29 (6)	Ag1^ii^—O9—Ag1	104.81 (5)
O4^v^—Ag2—O10	114.18 (6)	S2—O10—Ag1^ii^	123.30 (10)
O4—Ag2—O10	136.43 (6)	S2—O10—Ag1^iv^	118.98 (10)
O8—Ag2—O10	53.09 (5)	Ag1^ii^—O10—Ag1^iv^	108.58 (7)
O3—S1—O5	113.96 (11)	S2—O10—Ag2	97.40 (9)
O3—S1—O4	112.15 (11)	Ag1^ii^—O10—Ag2	121.23 (7)
O5—S1—O4	110.84 (11)	Ag1^iv^—O10—Ag2	79.11 (5)
O3—S1—C3	107.55 (11)	S2—O10—Ag2^ii^	67.58 (7)
O5—S1—C3	106.29 (11)	Ag1^ii^—O10—Ag2^ii^	59.96 (4)
O4—S1—C3	105.45 (11)	Ag1^iv^—O10—Ag2^ii^	166.05 (7)
O3—S1—Ag1	134.18 (8)	Ag2—O10—Ag2^ii^	113.18 (5)
O5—S1—Ag1	54.60 (8)	C2—C1—C6	120.7 (2)
O4—S1—Ag1	56.24 (7)	C2—C1—C7	121.0 (2)
C3—S1—Ag1	118.27 (8)	C6—C1—C7	118.3 (2)
O3—S1—Ag2	142.49 (8)	C3—C2—C1	119.0 (2)
O5—S1—Ag2	101.87 (8)	C3—C2—H2A	120.5
C3—S1—Ag2	70.67 (8)	C1—C2—H2A	120.5
Ag1—S1—Ag2	60.743 (12)	C2—C3—C4	120.7 (2)
O3—S1—Ag2^v^	75.89 (8)	C2—C3—S1	120.38 (19)
O5—S1—Ag2^v^	133.57 (8)	C4—C3—S1	118.86 (19)
C3—S1—Ag2^v^	113.59 (8)	C2—C3—Ag2	122.32 (16)
Ag1—S1—Ag2^v^	85.217 (16)	C4—C3—Ag2	67.59 (14)
Ag2—S1—Ag2^v^	71.389 (13)	S1—C3—Ag2	79.16 (8)
O3—S1—Ag2^iii^	89.34 (8)	C5—C4—C3	119.5 (2)
O4—S1—Ag2^iii^	105.55 (8)	C5—C4—Ag2	112.49 (17)
C3—S1—Ag2^iii^	135.36 (8)	C3—C4—Ag2	87.60 (15)
Ag1—S1—Ag2^iii^	58.742 (11)	C5—C4—H4	120.2
Ag2—S1—Ag2^iii^	118.903 (18)	C3—C4—H4	120.2
Ag2^v^—S1—Ag2^iii^	110.509 (17)	Ag2—C4—H4	70.2
O5—S1—Ag1^i^	110.81 (8)	C4—C5—C6	120.5 (2)
O4—S1—Ag1^i^	89.35 (7)	C4—C5—Ag2	47.98 (13)
C3—S1—Ag1^i^	131.60 (8)	C6—C5—Ag2	116.08 (17)
Ag1—S1—Ag1^i^	108.357 (17)	C4—C5—H5	119.7
Ag2—S1—Ag1^i^	127.793 (18)	C6—C5—H5	119.7
Ag2^v^—S1—Ag1^i^	56.515 (11)	Ag2—C5—H5	103.4
Ag2^iii^—S1—Ag1^i^	79.804 (14)	C5—C6—C1	119.4 (2)
O9—S2—O8	114.02 (11)	C5—C6—H6	120.3
O9—S2—O10	112.31 (11)	C1—C6—H6	120.3
O8—S2—O10	110.71 (11)	O1—C7—O2	124.1 (2)
O9—S2—C10	107.87 (11)	O1—C7—C1	121.7 (2)
O8—S2—C10	106.02 (11)	O2—C7—C1	114.2 (2)
O10—S2—C10	105.29 (11)	C13—C8—C9	120.9 (2)
O9—S2—Ag2	134.36 (8)	C13—C8—C14	120.9 (2)
O8—S2—Ag2	54.86 (8)	C9—C8—C14	118.2 (2)
O10—S2—Ag2	55.85 (8)	C10—C9—C8	118.7 (2)
C10—S2—Ag2	117.76 (8)	C10—C9—H9	120.6
O9—S2—Ag1^ii^	76.58 (8)	C8—C9—H9	120.6
O8—S2—Ag1^ii^	132.19 (8)	C9—C10—C11	120.9 (2)
C10—S2—Ag1^ii^	114.74 (8)	C9—C10—S2	120.11 (19)
Ag2—S2—Ag1^ii^	83.690 (15)	C11—C10—S2	118.95 (19)
O9—S2—Ag1^iv^	142.72 (8)	C9—C10—Ag1^iv^	122.76 (16)
O8—S2—Ag1^iv^	101.22 (8)	C11—C10—Ag1^iv^	67.27 (14)
C10—S2—Ag1^iv^	71.67 (8)	S2—C10—Ag1^iv^	78.38 (8)
Ag2—S2—Ag1^iv^	59.268 (11)	C12—C11—C10	119.3 (2)
Ag1^ii^—S2—Ag1^iv^	70.705 (13)	C12—C11—Ag1^iv^	111.71 (16)
O9—S2—Ag1	88.47 (8)	C10—C11—Ag1^iv^	88.31 (15)
O10—S2—Ag1	106.42 (8)	C12—C11—H11	120.4
C10—S2—Ag1	135.01 (8)	C10—C11—H11	120.4
Ag2—S2—Ag1	60.142 (11)	Ag1^iv^—C11—H11	70.3
Ag1^ii^—S2—Ag1	109.706 (17)	C11—C12—C13	120.7 (2)
Ag1^iv^—S2—Ag1	118.896 (18)	C11—C12—Ag1^iv^	48.75 (13)
O8—S2—Ag2^ii^	109.90 (8)	C13—C12—Ag1^iv^	116.17 (17)
O10—S2—Ag2^ii^	90.09 (7)	C11—C12—H12	119.6
C10—S2—Ag2^ii^	132.48 (8)	C13—C12—H12	119.6
Ag2—S2—Ag2^ii^	108.167 (17)	Ag1^iv^—C12—H12	102.7
Ag1^ii^—S2—Ag2^ii^	57.481 (11)	C12—C13—C8	119.4 (2)
Ag1^iv^—S2—Ag2^ii^	128.036 (18)	C12—C13—H13	120.3
Ag1—S2—Ag2^ii^	78.344 (13)	C8—C13—H13	120.3
C7—O2—H2	109.5	O6—C14—O7	124.4 (2)
S1—O3—Ag1^i^	135.48 (11)	O6—C14—C8	121.1 (2)
S1—O3—Ag2^v^	79.83 (8)	O7—C14—C8	114.6 (2)
			
C6—C1—C2—C3	1.7 (4)	C13—C8—C9—C10	1.5 (4)
C7—C1—C2—C3	−176.8 (2)	C14—C8—C9—C10	−178.8 (2)
C1—C2—C3—C4	−2.3 (4)	C8—C9—C10—C11	−3.1 (4)
C1—C2—C3—S1	175.60 (19)	C8—C9—C10—S2	173.79 (19)
C1—C2—C3—Ag2	79.1 (3)	C8—C9—C10—Ag1^iv^	78.3 (3)
O3—S1—C3—C2	98.3 (2)	O9—S2—C10—C9	98.0 (2)
O5—S1—C3—C2	−24.1 (2)	O8—S2—C10—C9	−24.5 (2)
O4—S1—C3—C2	−141.8 (2)	O10—S2—C10—C9	−141.9 (2)
Ag1—S1—C3—C2	−82.2 (2)	Ag2—S2—C10—C9	−82.8 (2)
Ag2—S1—C3—C2	−121.2 (2)	Ag1^ii^—S2—C10—C9	−178.88 (17)
Ag2^v^—S1—C3—C2	−179.76 (17)	Ag1^iv^—S2—C10—C9	−121.3 (2)
Ag2^iii^—S1—C3—C2	−9.3 (3)	Ag1—S2—C10—C9	−8.4 (3)
Ag1^i^—S1—C3—C2	114.91 (19)	Ag2^ii^—S2—C10—C9	113.49 (19)
O3—S1—C3—C4	−83.7 (2)	O9—S2—C10—C11	−85.1 (2)
O5—S1—C3—C4	153.9 (2)	O8—S2—C10—C11	152.4 (2)
O4—S1—C3—C4	36.1 (2)	O10—S2—C10—C11	35.0 (2)
Ag1—S1—C3—C4	95.7 (2)	Ag2—S2—C10—C11	94.1 (2)
Ag2—S1—C3—C4	56.71 (19)	Ag1^ii^—S2—C10—C11	−2.0 (2)
Ag2^v^—S1—C3—C4	−1.8 (2)	Ag1^iv^—S2—C10—C11	55.63 (18)
Ag2^iii^—S1—C3—C4	168.65 (14)	Ag1—S2—C10—C11	168.49 (14)
Ag1^i^—S1—C3—C4	−67.1 (2)	Ag2^ii^—S2—C10—C11	−69.6 (2)
O3—S1—C3—Ag2	−140.42 (9)	O9—S2—C10—Ag1^iv^	−140.70 (9)
O5—S1—C3—Ag2	97.16 (9)	O8—S2—C10—Ag1^iv^	96.78 (9)
O4—S1—C3—Ag2	−20.57 (9)	O10—S2—C10—Ag1^iv^	−20.60 (9)
Ag1—S1—C3—Ag2	39.04 (7)	Ag2—S2—C10—Ag1^iv^	38.49 (7)
Ag2^v^—S1—C3—Ag2	−58.52 (6)	Ag1^ii^—S2—C10—Ag1^iv^	−57.59 (6)
Ag2^iii^—S1—C3—Ag2	111.94 (8)	Ag1—S2—C10—Ag1^iv^	112.87 (8)
Ag1^i^—S1—C3—Ag2	−123.85 (7)	Ag2^ii^—S2—C10—Ag1^iv^	−125.22 (7)
C2—C3—C4—C5	0.7 (4)	C9—C10—C11—C12	1.6 (4)
S1—C3—C4—C5	−177.23 (19)	S2—C10—C11—C12	−175.29 (19)
Ag2—C3—C4—C5	−114.6 (2)	Ag1^iv^—C10—C11—C12	−114.1 (2)
C2—C3—C4—Ag2	115.3 (2)	C9—C10—C11—Ag1^iv^	115.7 (2)
S1—C3—C4—Ag2	−62.63 (17)	S2—C10—C11—Ag1^iv^	−61.23 (17)
C3—C4—C5—C6	1.5 (4)	C10—C11—C12—C13	1.5 (4)
Ag2—C4—C5—C6	−99.0 (2)	Ag1^iv^—C11—C12—C13	−99.3 (2)
C3—C4—C5—Ag2	100.5 (3)	C10—C11—C12—Ag1^iv^	100.8 (3)
C4—C5—C6—C1	−2.1 (4)	C11—C12—C13—C8	−3.0 (4)
Ag2—C5—C6—C1	−56.9 (3)	Ag1^iv^—C12—C13—C8	−58.8 (3)
C2—C1—C6—C5	0.5 (4)	C9—C8—C13—C12	1.5 (4)
C7—C1—C6—C5	179.0 (2)	C14—C8—C13—C12	−178.2 (2)
C2—C1—C7—O1	163.7 (3)	C13—C8—C14—O6	155.1 (2)
C6—C1—C7—O1	−14.8 (4)	C9—C8—C14—O6	−24.6 (4)
C2—C1—C7—O2	−16.4 (3)	C13—C8—C14—O7	−25.5 (3)
C6—C1—C7—O2	165.0 (2)	C9—C8—C14—O7	154.8 (2)

Symmetry codes: (i) −*x*+3, −*y*+1, −*z*+1; (ii) −*x*+2, −*y*, −*z*+1; (iii) *x*+1, *y*, *z*; (iv) *x*−1, *y*, *z*; (v) −*x*+2, −*y*+1, −*z*+1.

### Hydrogen-bond geometry (Å, º)

**Table:**

*D*—H···*A*	*D*—H	H···*A*	*D*···*A*	*D*—H···*A*
O2—H2···O1^vi^	0.84	1.81	2.631 (3)	164
O7—H7···O6^vii^	0.84	1.81	2.651 (3)	176
C4—H4···O8^v^	0.95	2.43	3.214 (3)	140
C11—H11···O5^ii^	0.95	2.48	3.269 (3)	141

Symmetry codes: (ii) −*x*+2, −*y*, −*z*+1; (v) −*x*+2, −*y*+1, −*z*+1; (vi) −*x*+2, −*y*, −*z*; (vii) −*x*+2, −*y*+1, −*z*+2.
